# A Randomized-Controlled Trial Pilot Study Examining the Effect of Pelvic Floor Muscle Training on the Irisin Concentration in Overweight or Obese Elderly Women with Stress Urinary Incontinence

**DOI:** 10.1155/2019/7356187

**Published:** 2019-08-19

**Authors:** Magdalena Weber-Rajek, Agnieszka Radzimińska, Agnieszka Strączyńska, Katarzyna Strojek, Zuzanna Piekorz, Mariusz Kozakiewicz, Hanna Styczyńska

**Affiliations:** ^1^Department of Physiotherapy, Collegium Medicum in Bydgoszcz, Nicolaus Copernicus University in Torun, Poland; ^2^Department of Food Chemistry, Collegium Medicum in Bydgoszcz, Nicolaus Copernicus University in Torun, Poland

## Abstract

**Objective:**

This study aimed to examine the effect of pelvic floor muscle training on the irisin (Ir) concentration in overweight or obese elderly women with stress urinary incontinence.

**Methods:**

The number of participants included in analysis was 49: 28 women in the experimental group and 21 women in the control group. The experimental group (EG) underwent pelvic floor muscle training, whereas no therapeutic intervention was applied to the control group (CG). Irisin concentration, severity of urinary incontinence (RUIS), and body mass index (BMI) were measured in all women at the initial and final assessments.

**Results:**

By comparing the initial and final assessment results we have been able to demonstrate statistically significant differences in the measured variables in the experimental group. No statistically significant differences in the measured variables were reported for the control group at the initial and final assessments. Moderate negative correlation was observed between the BMI results with the irisin concentration results in the EG at the initial assessment and no correlation at the final assessment. Weak positive correlation was observed between the BMI results with the irisin concentration in the CG at the initial and final assessment.

**Conclusion:**

Further studies are necessary to observe the regulation of irisin concentration and explain mechanisms underlying these effects.

## 1. Introduction

Stress urinary incontinence (SUI) is the most common type of urinary incontinence (UI). Atrophy and impairment of type II fibers in the levator ani muscles play an essential role in the development of SUI in women. Type II to type I muscle fiber switching is a beneficial physiological mechanism induced by skeletal muscle adaptation to exercise. However, if the process occurs locally, close to the urethral sphincter, then it has a negative effect in the form of SUI. The dysfunction of suspensory ligaments of the urethra and/or reduced contractility of the sphincter urethrae due to myofascial dysfunction of the pelvic floor are also associated with stress urinary incontinence in women [1, 2].

We distinguish diverse types of risk factors for urinary incontinence: predisposing, decompensating, and promoting. Among these there are certain risk factors for both UI and obesity, such as a woman's age, environmental diseases, level of physical activity, diet, and occurrence of menopause. Obesity is conducive to the occurrence of urinary incontinence; however, it can also increase the severity of this condition [3]. Targeted physical activity plays a critical role in the treatment of UI and obesity. The muscle is no longer seen as a simple contractile motor, but as crossroads of more complex networks, involving a reduction of protein (synthesis and regeneration), with a parallel increase of apoptosis and protein-lysis. [4–6]. Proteins secreted by muscle fibres (myocytes) are known as myokines. The following myokines are secreted by muscle cells in response to muscle contraction: angiopoietin-like protein 4 (ANGPTL4), fibroblast growth factor 21 (FGF21), interleukin 6 (IL-6), interleukin 7 (IL-7), interleukin 15 (IL15), myonectin (CTRP15), myostatin (MSTN), vascular endothelial growth factor (VEGF), follistatin (FST), and irisin (Ir) [7–11].

Irisin was described for the first time in 2012 by Boström et al. [12] as a new myokine which results from the cleavage of the type I membrane protein, fibronectin type III containing five domains (FNDC5). This cleavage is induced by the peroxisome proliferator-activated receptor *γ* (PPARy) transcriptional coactivator PGC-1*α*. Irisin is secreted from FNDC5, and the process itself is regulated by physical activity and stimulation of PGC-1*α* (one of the most important energy metabolism regulators). Increase of PGC-1*α* expression is accompanied by mitochondrial biogenesis, increase of insulin dependent glucose uptake, increase of the neuromuscular connections, and angiogenesis stimulation. Thus, the PGC-1*α* expression plays a critical role in maintaining carbohydrate, lipid, and energy homeostasis in the body [13–15].

## 2. Aim of the Study

This study aimed to examine the effect of pelvic floor muscle training on the irisin concentration in overweight or obese elderly women with stress urinary incontinence.

## 3. Methods

### 3.1. Study Design

Between January 2017 and May 2017, 62 women suffering from UI were enrolled into a randomized, double-blind, controlled study. The following was conducted in accordance with the principles of the Declaration of Helsinki. The authors of this study obtained an approval from the Local Bioethics Committee in Poland. Moreover, a statement confirming written informed consent was obtained from all the participants All deidentified data included in this are contained within this report. In order to ensure stratified randomization, the researchers allocated the subjects using a rather simple method. Specifically, envelopes with group allocation numbers were picked from a computer generated random number table [16]. The main investigator was blinded throughout the group allocation. The initial number of patients assessed for eligibility was 98. Of 36 women who were excluded in the first stage of the study: 34 participants did not meet the inclusion criteria, whereas 2 participants refused to participate. Subsequently, 62 women were randomly assigned to the experimental group (EG) who underwent pelvic floor muscle training (PFMT) or the control group (CG).

All women in EG group underwent 12 therapy sessions (3 times a week, 45-minute sessions, lasting 4 weeks) according to the proprietary program. The exercises were completed in five/six-person groups supervised by a physiotherapist. Before joining the training session, all patients were examined for posture and body correction and then prepared for a proper training by acquiring the skills of mobilising sacroiliac joints and doing exercises to improve the range of movement in the lumbar-sacral spine and the hip and knee joints. Training in breathing through abdominal and thoracic duct was also conducted. The Pelvic Floor Muscle Training (PMFT) program was based on straining fast and slow twitch muscle fibers of the pelvic floor with relaxed gluteal muscles using the transversus abdominis muscle tension, without changing the position and with changing the position as well as synchronising the muscles with breathing. The PFMT exercise was performed in lying, sitting, and standing positions. The numbers of exercises and repetitions were determined individually depending on the functional abilities in patients. However, no therapeutic intervention was applied to the control group (CG).

**Figure 1:**
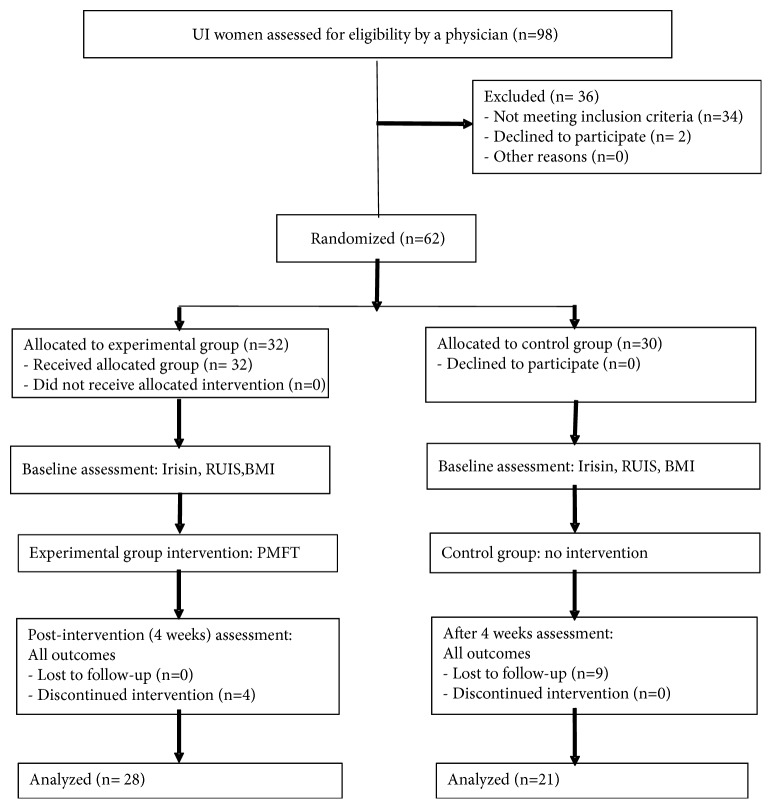
The study flow diagram.

Of 13 participants who did not complete the study 4 participants withdrew from the EG during the 4-week intervention program, whereas 9 participants from the CG missed the final study visit. Overall, 49 participants have successfully completed the study (EG n = 28; CG n = 21). The randomized control trials (RCT) reporting quality have been improved using the CONSORT statement (Consolidated Standards of Reporting Trials) (Figure 1) [17].

Before the treatment, all the women were asked about the circumstances of urine loss, and the presence of comorbid conditions and contraindications to the treatment. Additionally, the Questionnaire for Urinary Incontinence Diagnosis (QUID) was used to diagnose the UI type. The QUID is a 6-item UI symptom questionnaire developed and validated to distinguish stress and urge urinary incontinence. Since the QUID includes acceptable psychometric characteristics, it can be used as a UI outcome measure in clinical trials [18].

Study inclusion criteria were as follows: age 60 years or older, a Body Mass Index (BMI) of 25 or more, diagnosed SUI, and no contradictions to the treatment (uterine tumors and myomas, urinary or genital tract infections, acute inflammations, acute infections, grade 3 or 4 hemorrhoids, stage 3 uterine prolapse, recent pelvic fractures, recent surgeries, severe urethral sphincter weakness and/or defect, suspected urethral and/or vesical fistula).

Study exclusion criteria were as follows: age < 60, a BMI under 25, diagnosed urge and mixed urinary incontinence, lack of regular physical activity, no therapeutic interventions in UI in the last three months (PFMT, Extracorporeal Magnetic Innervation (ExMI), electrostimulation, biofeedback), and the presence of contraindications to the treatment.

### 3.2. Measurements

To objectify the results, irisin concentrations were obtained for the EG and the CG at the initial and final assessments. Moreover, urinary incontinence severity assessment results (RUIS) and body mass index (BMI) were recorded for each study participant.

### 3.3. Irisin Concentration

Vacuette tubes with EDTA anticoagulant were used to draw 6 ml of fasting blood from each participant. The authors analyzed the samples using a competitive enzyme-linked immunosorbent assay (ELISA) (BIOVENDOR IRISIN ELISA kit, cat. no.: RAG018R, Brno, Czech Republic). The purified antigen competes with the antigen in the test sample for binding to an antibody that has been immobilized in a microtiter plate. This method is used for quantitative determination of irisin in human plasma. A polyclonal antibody recognizing native irisin reacts with a series of predetermined recombinant irisin standard proteins or samples under competition in the irisin-coated plate. Absorbance was measured at 450 nm in an ELISA reader. The standard curve is generated by plotting the average absorbance obtained for each standard concentration vs the corresponding irisin concentration (*μ*g/ml), whereas irisin concentration in samples was calculated using the interpolation of the regression curve formula of a 4-parameter logistic equation.

### 3.4. The Revised Urinary Incontinence Scale (RUIS)

The RUIS is a valid 5-item scale that may be used to assess UI and to monitor patient outcomes after the treatment. A score of 0–3 is considered non-urinary incontinence; 4–8 mild urinary incontinence; 9–12 moderate urinary incontinence, and 13 or above severe urinary incontinence [19].

*Body Mass Index (BMI)* is determined as body mass in kilograms divided by height in meters squared. For adults, the BMI categories are as follows: < 18.5 underweight; 18.5–24.99 normal weight; ≥ 25.0 overweight.

### 3.5. Intervention

The experimental group underwent 12 therapy sessions of PFMT (45 minutes each, 3 times a week during 4 weeks).

### 3.6. Statistical Analyses

The collected data was analyzed statistically using the Statistica 13.1 software. Lower quartile (Q1), upper quartile (Q3), and the median were measured. Differences between the two groups were estimated using the Mann–Whitney U-test. Differences within one group were estimated using the Wilcoxon test. The correlation between measured variables was checked using the Spearman correlation coefficient. The authors defined statistical significance level as p < 0.05.

## 4. Results

Table 1 presents Student's t-test results and descriptive statistics for all measured variables for the EG and CG at the initial assessment. After comparing the Mann–Whitney U-test p value with the significance level of *α* = 0.05, the authors found no statistically significant differences between the experimental group and the control group results at the initial assessment. This confirms the homogeneity of the study groups.

Table 2 presents Wilcoxon test results and descriptive statistics for all measured variables for the EG and CG at the initial and final assessments.

After comparing the Wilcoxon test p value with the significance level of *α* = 0.05, the authors found a statistically significant difference in all measured variables for the experimental group at the initial and final assessments. A statistically significant increase in irisin concentration, decreases in BMI result, and an improvement in severity of UI (RUIS) were recorded for the PFMT group at the final assessment. After comparing the Wilcoxon test p value with the significance level of *α* = 0.05, the authors found no statistically significant differences between all measured variables for the control group at the initial and final assessments.

Table 3 presents the relationship between the irisin concentration assessment for the EG at the initial and final assessments and the UI severity (RUIS).

After comparing the Wilcoxon test p value with the significance level of *α* = 0.05, the authors found a statistically significant difference in irisin concentration at the final assessment in patients with severe, moderate, and mild urinary incontinence.

Table 4 presents Mann–Whitney U-test results and descriptive statistics for all measured variables for the EG and CG at the final assessment.

After comparing the Mann–Whitney U-test p value with the significance level of *α* = 0.05, the authors found no statistically significant difference in the measured variables between the experimental group and the control group at the final assessment.

In the last stage of the study, we correlated the BMI results with the irisin concentration results recorded for the experimental and control groups at the initial and final assessment. Moderate negative correlation was observed between the measured variables in the experimental group at the initial assessment (r = - 0. 435) and no correlation at the final assessment (r = - 0.187), whereas weak positive correlation was observed between the measured variables in the control group at the initial assessment (r = 0. 219) and at the final assessment (r = 0.207).

**Figure 2:**
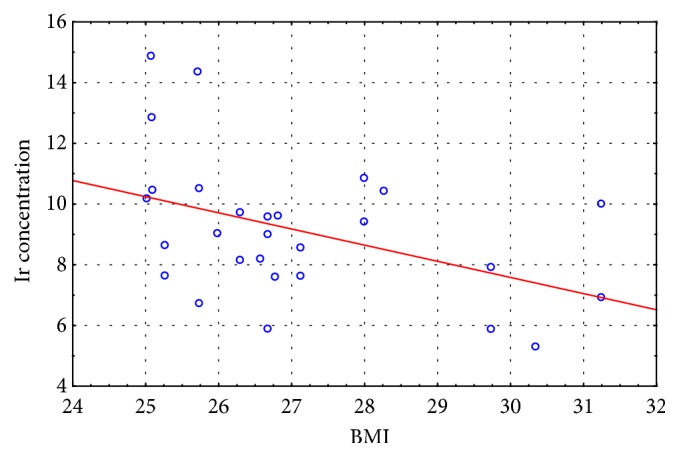
The scatter diagram: BMI to Ir concentration at the initial assessment for the experimental group.

**Figure 3:**
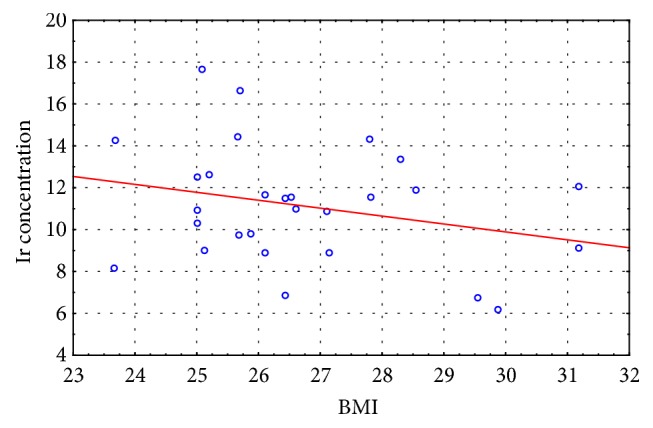
The scatter diagram: BMI to Ir concentration at the final assessment for the experimental group.

**Figure 4:**
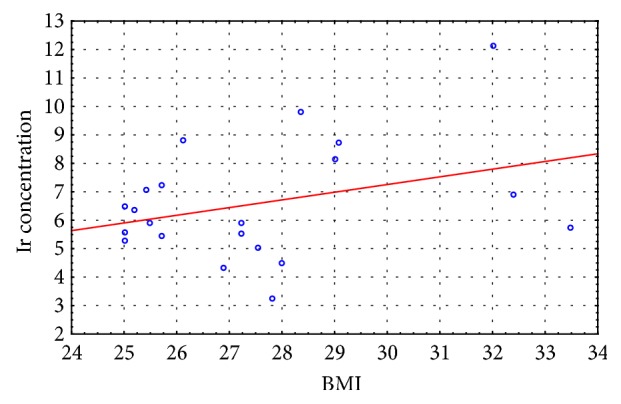
The scatter diagram: BMI to Ir concentration at the initial assessment for the control group.

**Figure 5:**
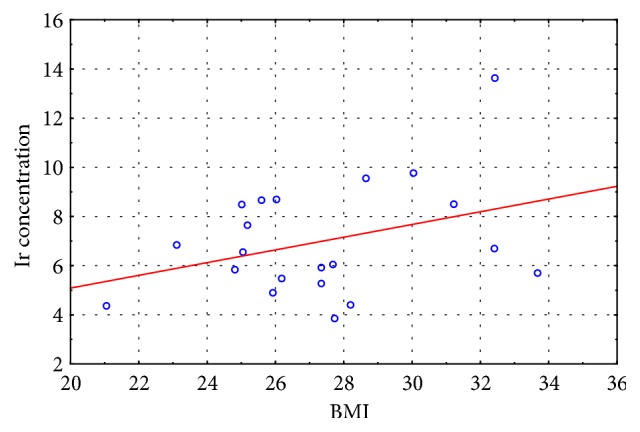
The scatter diagram: BMI to Ir concentration at the final assessment for the control group.

The scatter diagrams (Figures 2–5) show the association between BMI and the irisin concentration at the initial assessment and at the final assessment for the experimental and control groups.

## 5. Discussion

To our knowledge, this is the first study which assesses irisin concentration after PFMTin overweight or obese elderly women with stress urinary incontinence. Valid question remains how the physical activity intensity (chronic or acute) affects irisin concentration. The authors attempted to answer this question by evaluating this myokine concentration after a chronic physical activity, i.e., a 4-week pelvic floor muscle training. Furthermore, in this study the authors assessed the Body Mass Index and the severity of urinary incontinence using the Revised Urinary Incontinence Scale (RUIS). By comparing the results at the initial and final assessments, we found a statistically significant increase in the irisin concentration for the EG (p < 0.001) and no statistically significant differences in the irisin concentration in the CG (p=0.079). Moreover, by comparing the results at the initial and final assessments, we found a statistically significant improvement in severity of urinary incontinence for the EG (p < 0.001) and no statistically significant differences in severity of UI in the CG (p=0.124). Even more interestingly, we recorded a statistically significant increase in irisin concentration in experimental group patients with mild, moderate, and severe urinary incontinence at the final assessment.

The Boström et al. study, published in 2012 [12], showed an increase in plasma irisin in eight healthy men following a 10-week aerobic training and afterwards it sparked a discussion about factors that affect myokine expression. Among such factors are age [20, 21], physical activity type [22–25], training level [23, 26, 27], anthropometric parameters [26, 28], comorbid diseases [29–31], and study design for measuring Ir concentration [32–34].

Since the study participants were overweight or obese women (BMI > 25), we also evaluated the BMI results for the EG and CG at the initial and final assessments. By comparing the results at the initial and final assessments, the authors found a statistically significant decrease in BMI results in EG (p < 0.001) and no statistically significant differences in the BMI results in the CG (p=0.754).

Because irisin concentration impacts body's metabolic profile, the authors also carried out studies to evaluate correlations between BMI results and the Ir concentration both in the EG and CG at the initial and final assessments. Moderate negative correlation was observed between the measured variables in the experimental group at the initial assessment (r = - 0. 435) and no correlation at the final assessment (r = - 0.187), whereas weak positive correlation was observed between the measured variables in the control group at the initial assessment (r = 0. 219) and at the final assessment (r = 0.207). The correlation between the irisin concentration and the BMI score is not clearly understood given that published studies offer conflicting results regarding this matter (positive correlation, negative correlation, and no correlation) [15, 29, 35–37]. Roca-Rivada et al. [37] demonstrated for the first time that white adipose tissue (WAT) also secretes FNDC5, and it can behave as an adipokine, constituting about 28% of the circulating irisin level in the blood. Research conducted using rat adipose tissue explants showed that visceral adipose tissue (VAT), and specifically subcutaneous adipose tissue (SAT), expresses and secretes FNDC5. During physical exertion the muscle tissue affects the circulating protein level, whereas in the case of obesity it is the fat tissue that actively increases the Ir concentration. Furthermore, greater skeletal muscle mass, which is a strong predictor of Ir, can also explain the positive correlation between BMI result and irisin concentration [26], while the hypotheses regarding the negative correlation of BMI and irisin concentration are associated with brown adipose tissue (BAT). Adipose tissue is heterogeneous. White adipose tissue (WAT) stores energy and its distribution greatly affects metabolic risk, whereas brown adipose tissue burns energy for thermogenesis. BAT is present and can be activated in most adult humans and total BAT activity is inversely associated with adiposity and indexes of the metabolic syndrome [38]. In overweight and obesity have been described significant low amounts of BAT, which confirms the irisin-resistance hypothesis [39, 40].

## 6. Conclusions

1. The therapeutic program induced a statistically significant increase in irisin concentration in the study participants.
2. The therapeutic program improved the severity of urinary incontinence in the study participants.
3. There was moderate negative correlation between BMI results and irisin concentration in the experimental group.
4. There was weak positive correlation between BMI results and irisin concentration in the control group.
5. Further studies are necessary to observe the regulation of irisin concentration and explain mechanisms underlying these effects.

## Figures and Tables

**Table 1:** Comparative analysis of all measured variables for the EG and CG – the initial assessment.

Parameter	Statistics	EG (n=28)	CG (n=21)	p value
*Age*	IQR	2.00	6.00	0.630
	Med	62.50	67.00	

*BMI* (kg/m^2^)	IQR	2.40	2.85	1.000
	Med	26.67	27.22	

*Irisin concentration* (ng/ml)	IQR	2.67	1.77	1.000
	Med	9.02	5.91	

*RUIS* (points)	IQR	6.00	3.00	1.000
	Med	10.00	7.00	

EG – experimental group; CG – control group; Med – median; IQR – interquartile range;

p – significance level; RUIS – Revised Urinary Incontinence Scale; BMI-Body Mass Index

**Table 2:** Comparative analysis of all measured variables for the EG and CG – at the initial and final assessments.

Parameter	Statistics	EG (n=28)		p value	CG (n=21)		p value
		Initial assessment	Final assessment		Initial assessment	Final assessment	
*Irisin concentration* (ng/ml)	IQR	2.67	3.49	< 0.001 *∗*	1.77	3.01	0.079
	Med	9.02	11.19		5.91	6.55	

*RUIS* (points)	IQR	6.00	5.50	< 0.001 *∗*	3.00	3.00	0.124
	Med	10.00	7.00		7.00	6.00	

*BMI* (kg/m^2^)	IQR	2.40	2.64	< 0.001 *∗*	2.85	3.47	0.754
	Med	26.67	26.28		27.22	27.34	

EG – experimental group; CG – control group; Med – median; IQR – interquartile range; p – significance level; RUIS – Revised Urinary Incontinence Scale; BMI – Body Mass Index, *∗* statistical significance

**Table 3:** The relationship between the assessment of irisin concentration for the EG at the initial and final assessments and RUIS.

RUIS (points)	N	Statistics	Irisin concentration (ng/ml)		p value
			Initial assessment	Final assessment	
Mild	9	IQR	2.86	3.42	0.007*∗*
		Med	9.01	10.94	

Moderate	11	IQR	2.54	5.13	0.003*∗*
		Med	8.20	10.27	

Severe	8	IQR	3.74	4.73	0.011**∗**
		Med	10.23	12.24	

EG – experimental group; Med – median; IQR – interquartile range; N – number of participants;

p – significance level; RUIS – Revised Urinary Incontinence Scale; *∗*statistical significance

**Table 4:** Comparative analysis of all measured variables for the EG and CG – the final assessment.

Parameter	Statistics	EG (n=28)	CG (n=21)	p value
*Irisin concentration* (ng/ml)	IQR	3.49	3.01	1.000
	Med	11.19	6.55	

*RUIS* (points)	IQR	5.50	3.00	0.215
	Med	7.00	6.00	

*BMI*	IQR	2.64	3.47	1.000
	Med	26.28	27.34	

EG – experimental group; CG – control group; Med – median; IQR – interquartile range; p – significance level;

RUIS – Revised Urinary Incontinence Scale; BMI – Body Mass Index

## Data Availability

The data used to support the findings of this study are available from the corresponding author upon request.

## Additional Points

*Study Limitations*. The authors acknowledge certain limitations of their analysis. These include a relatively small study group and the lack of assessment of long-term treatment outcomes. We therefore consider this research as a pilot study and we plan to further study this topic.
